# Organ-Specific Metabolic Shifts of Flavonoids in *Scutellaria baicalensis* at Different Growth and Development Stages

**DOI:** 10.3390/molecules23020428

**Published:** 2018-02-15

**Authors:** Jingyuan Xu, Yilan Yu, Ruoyun Shi, Guoyong Xie, Yan Zhu, Gang Wu, Minjian Qin

**Affiliations:** Department of Resources Science of Traditional Chinese Medicines, School of Traditional Chinese Pharmacy and State Key Laboratory of Natural Medicines, China Pharmaceutical University, Nanjing 210009, China; Xujingyuanxu@126.com (J.X.); 15250962227@163.com (Y.Y.); spajk3245@126.com (R.S.); guoyongxie321@163.com (G.X.); cpuzy@126.com (Y.Z.); woosmail@163.com (G.W.)

**Keywords:** *Scutellaria baicalensis*, dynamic accumulation, organ-specific, growth, metabolic profile

## Abstract

*Scutellaria baicalensis* Georgi is a traditional Chinese herbal medicine mainly containing flavonoids that contribute to its bioactivities. In this study, the distributions and dynamic changes of flavonoid levels in various organs of *S. baicalensis* at different development stages were investigated by UHPLC-QTOF-MS/MS and HPLC-DAD methods. The results indicated that the metabolic profiles of *S. baicalensis* changed with growth and development. During the initial germination stage, the seeds mainly contained flavonols. With growth, the main kinds of flavonoids in *S. baicalensis* changed from flavonols to flavanones and flavones. The results also revealed that the accumulation of flavonoids in *S. baicalensis* is organ-specific. The flavones without 4′-OH groups mainly accumulate in the root and the flavanones mainly accumulate in aerial organs. Dynamic accumulation analysis showed that the main flavonoids in the root of *S. baicalensis* accumulated rapidly before the full-bloom stage, then changed to a small extent. The results suggested the proper harvest time for the aerial parts was at the initial stage of reproductive growth and the flower buds should be collected before flowering. This study deepening the knowledge of *S. baicalensis* should provide valuable information for guiding the scientific cultivation of this plant and the development and utilization of *S. baicalensis*.

## 1. Introduction

*Scutellaria baicalensis* Georgi is a medicinal plant of the Lamiaceae family. Its roots have been used as a traditional Chinese medicine (known as *Huang Qin* in Chinese), for the treatment of many diseases such as influenza, pneumonia, and dysentery [1,2] for over 2000 years. Many studies have demonstrated that *Huang Qin* possesses a wide range of pharmacological effects, including antitumor, hepatoprotective and antibacterial effect [3,4,5]. It is believed that flavones, such as baicalin, wogonoside and their corresponding aglycones baicalein and wogonin are the major bioactive substances responsible for its reliable and efficacious biological activities [6]. 

On account of its important medicinal value, the annual demand for *S. baicalensis* is increasing year by year. The yield of *S. baicalensis* based on wild sources is nowadays insufficient and it is gradually being replaced by artificially cultivated plants. As the aerial parts (including the stem, leaf and flower) of *S. baicalensis* are not the traditional medicinal parts, they are usually discarded as waste during the root harvesting or only used as a functional food in limited areas [7], which results in a significant waste of resources. In recently years, researches on the aerial parts of *S. baicalensis* have attracted more and more attentions. Modern pharmacological studies have shown that the aerial parts of *S. baicalensis* also have biological activities, such as antiviral [8], anti-lipid peroxidation [9], neuroprotective [10] and cardiocyte protective activity [11]. The main bioactive constituents in the aerial parts of *S. baicalensis* are also flavonoids [7,12], such as scutellarin [13], apigenin-7-*O*-*β*-d-glucuronide [14] and baicalin. Therefore, it is worthwhile to exploit the aerial parts of *S. baicalensis*. 

Until now, the identifications of flavonoids in *S. baicalensis* have been reported by several research groups [6,12,15]. However, there is no report about the metabolic profiles of all the growth and development stages in the whole life cycle of *S. baicalensis*. The dynamic changes of flavonoid levels in various organs of *S. baicalensis* at different growth and development stages remain unknown. During the plant growth and development, tissue specialization is never an independent event. It is an intricate biological process where differentiation at the physical, chemical, and biological levels, takes place simultaneously [16]. These changes result in a series of dynamic modifications to the whole metabolic pathway network. Understanding these changes could help us to determine the possible physiological effects of metabolites in the plant and guide us to cultivate and utilize the medicinal plant resource in a reasonable manner.

In the present study, the metabolic profiles of *S. baicalensis* from seed germination to the reproductive growth stage were analyzed by ultra-high-performance liquid chromatography coupled to quadrupole-time of flight mass spectrometry (UHPLC-QTOF-MS/MS). The contents of the main flavonoids in different organs of *S. baicalensis* collected at different growth and development stages were further quantitatively analyzed. This work provides whole metabolic profiles for the complete life cycle of *S. baicalensis*. Meanwhile, the accumulation patterns of main flavonoids in the different organs of *S. baicalensis* at different development periods were also studied. This could effectively promote understanding of how the flavonoid metabolism in *S. baicalensis* changes and provide us valuable information for the scientific cultivation and optimum harvesting of *S. baicalensis*.

## 2. Results and Discussion

### 2.1. The Metabolic Profiles of S. baicalensis Were Organ-Specific and Changed at Different Growth and Development Stages

**Figure 1:**
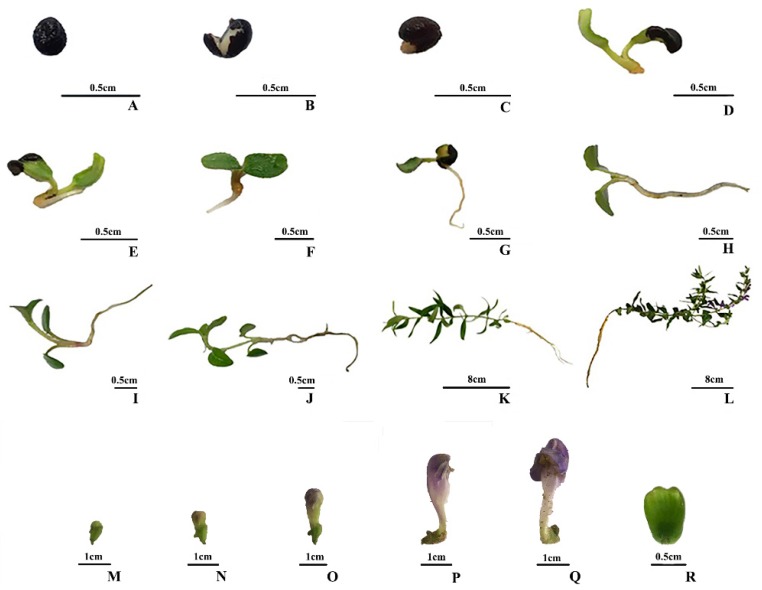
Samples collected at different growth stages. (**A**): sample collected at the beginning of sowing (day 0); (**B**): sample collected at the second day (day 2); (**C**): sample collected at the fourth day (day 4); (**D**): sample collected at the 6th day (day 6); (**E**) sample collected at the 8th day (day 8); (**F**): sample collected at the 10th day (day 10); (**G**): sample collected at the 12th day (day 12); (**H**): sample collected at the 14th day (day 14); (**I**): sample collected at the 18th day (day 18); (**J**): sample collected at the 22th day (day 22); (**K**): sample collected at the 62th day (day 62); (**L**): sample collected at the 83th day (day 83); (**M**): flower bud 1 (FB1); (**N**): flower bud 2 (FB2); (**O**): flower bud 3 (FB3); (**P**): flower bud 4 (FB4); (**Q**): flower (FL); (**R**): fruit (FR).

Previous studies showed that *S. baicalensis* contained a large number of flavonoids [2], but it was not clear how the metabolic profiles of *S. baicalensis* changed at different growth and development stages. To reveal the dynamic changes of metabolic profiles in various organs of *S. baicalensis* at different growth and development stages, samples were collected from the beginning of seed sowing to fruiting (Figure 1). 

According to the phenotypic difference, the samples were divided into three stages which were the seed germination stage (from day 0 to day 8), seedling stage (from day 10 to day 22) and plant maturity stage (day 62 and day 83), respectively. The collected samples were analyzed by UHPLC-QTOF-MS/MS. A total of 98 different compounds were characterized in 27 different samples (Table 1). The identified compounds were mainly flavonoids, while some other compounds classes such as phenylpropanoid glycosides, phenolic acids and quinones were also authenticated in *S. baicalensis* (Figure S1).

**Figure 2:**
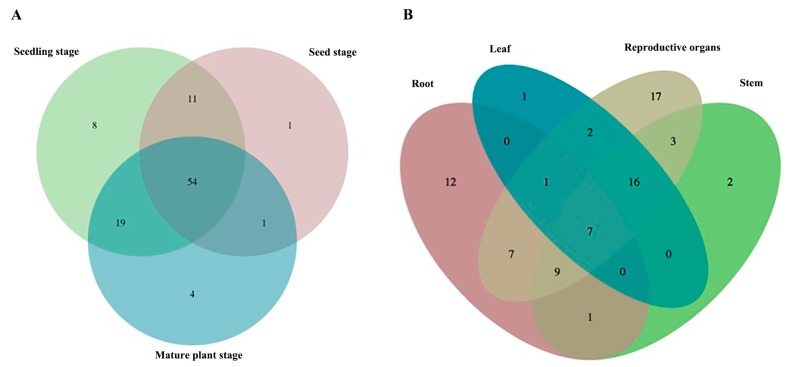
Venn diagrams of identified metabolites in *S. baicalensis*. (**A**) was the distribution of unique metabolites and common metabolites in *S. baicalensis* at seed stage, seedling stage and mature plant stage. (**B**) was the distributions of unique metabolites and common metabolites in root, leaf, stem and reproductive organs of *S. baicalensis*.

At the seed germination stage, the main metabolites were flavonols, such as dihydromyricetin-3′-*O*-glucoside, taxifolin-7-*O*-glucoside, dihydromyricetin, kaempferol-3-*O*-glucoside and their isomers. They might mainly exist in the episperm and participate in defending against pathogens and UV-damage [34,35]. With seed germination and seedling growth, the metabolic profiles of *S. baicalensis* changed. The flavonols did not accumulate with growth, but rather flavones such as scutellarin, baicalin and chrysin-7-*O*-*β*-d-glucuronide began to accumulate. The numbers of identified compounds which were unique in each stage were one for the seed germination stage, eight for the seedling stage and four for the mature plant stage (Figure 2A) and they are listed in Table 2. The results demonstrated that flavonoids were synthesized at each growth stage of *S. baicalensis*, but the metabolic profiles of different development stages obviously differed. 

The results of the metabolic analysis also showed that the types of identified compounds in different organs were in the order: reproductive organs > stem > root > leaf. The four parts shared seven common compounds, and the number of unique identified compounds that could only be found in each part were 12 for the roots, two for the stems, one for the leaves and 17 for the reproductive organs (Figure 2B and Table 2). The main type of flavonoids in the root were flavonoids without a 4′-OH group, which were significantly different from those with 4′-OH in the aerial organs. This might be due to a recently discovered evolved pathway for biosynthesis of specific 4′-deoxyflavones in the roots of *S. baicalensis* [36]. Therefore, the results indicated that in *S. baicalensis*, the metabolic profiles of different aerial organs were similar and they were different from those of the roots.

**Figure 3:**
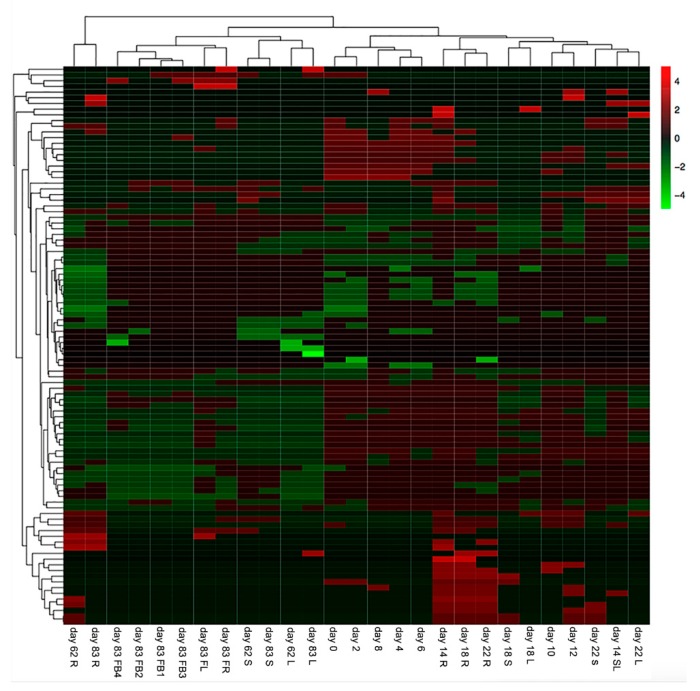
Heat map of the identified metabolites in different organs of *S. baicalensis* during different growth stages. R: the root of *S. baicalensis*; S: the stem of *S. baicalensis*; L: the leaves of *S. baicalensis;* SL: the leaves and stems of *S. baicalensis*; FB1~FB4: the flower bud 1~flower bud 4 of *S. baicalensis*; FL: the flowers of *S. baicalensis*; Fr: the fruits of *S. baicalensis*.

According to the distribution of the characterized compounds, the samples could also be divided into different clusters by the unweighted pair-group method with arithmetic (UPGMA) clustering method (Figure 3). 

The samples collected at the mature plant stage were distinguished from the samples collected at the seed germination stage and seedling stage. Both the roots of *S. baicalensis* collected at the 62th day (day 62 R) and the roots of *S. baicalensis* collected at the 83th day (day 83 R) were clustered into the first branch and significantly separated from other samples. The aerial parts of the samples at mature plant stage were separated into two branches. The reproductive organs collected on the 83th day were clustered into the second branch and the vegetative organs were clustered into the third branch. The fourth branch grouped samples collected from the seed germination stage. The samples collected from the seedling stage were clustered into the fifth branch and they had a huge difference in morphology compared with the samples in the fourth branch. The above results also indicated that the metabolic profiles of *S. baicalensis* were organ-specific and changed with the growth and development phases. 

### 2.2. Dynamic Accumulation of Main Flavonoids in S. baicalensis from Seed Sowing to the True Leaf Coming

**Figure 4:**
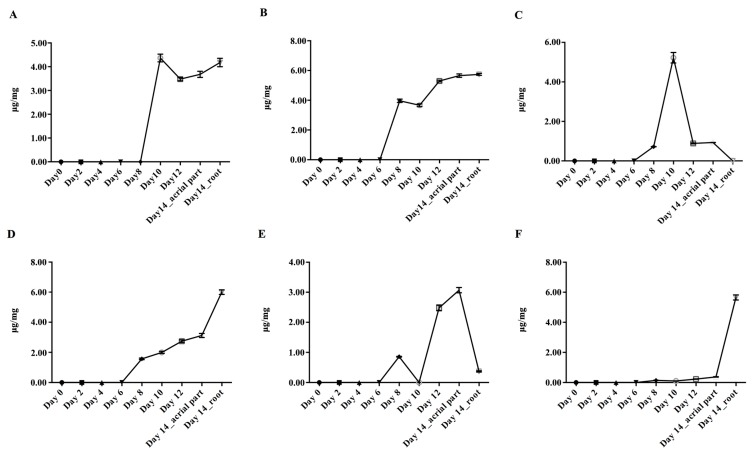
The accumulation patterns of flavonoids in *S. baicalensis* from seed germination to the true leaves coming out. (**A**): Isocarthamidin-7-*O*-*β*-d-glucuronide; (**B**): Scutellarin; (**C**): Apigenin-7-*O*-*β*-d-glucuronide; (**D**): Baicalin; (**E**): Chrysin-7-*O*-*β*-d-glucuronide; (**F**): Wogonoside; The units “μg/mg” meant the contents of analytes in per mg of freeze-dried samples.

The results of method validation are presented in Table S1. As shown in Figure 4, from the beginning of seed sowing (day 0) to the 6th day (day 6), the flavonoids which mainly existed in the mature plant of *S. baicalensis* were too low to be quantified. After the 6th day of seed sowing, the contents of isocarthamidin-7-*O*-*β*-d-glucuronide, scutellarin, apigenin-7-*O*-*β*-d-glucuronide, baicalin, chrysin-7-*O*-*β*-d-glucuronide and wogonoside gradually increased. By the 14th day, when the true leaves appeared, the samples were separated into two groups, the aerial parts and roots. The contents of baicalin and wogonoside in roots were higher than those in the aerial parts. The contents of isocarthamidin-7-*O*-*β*-d-glucuronide and scutellarin in roots were similar to those in aerial parts. Apigenin-7-*O*-*β*-d-glucuronide and chrysin-7-*O*-*β*-d-glucuronide mainly existed in the aerial parts.

### 2.3. The Accumulation Patterns of Main Flavonoids in Roots of S. baicalensis

**Figure 5:**
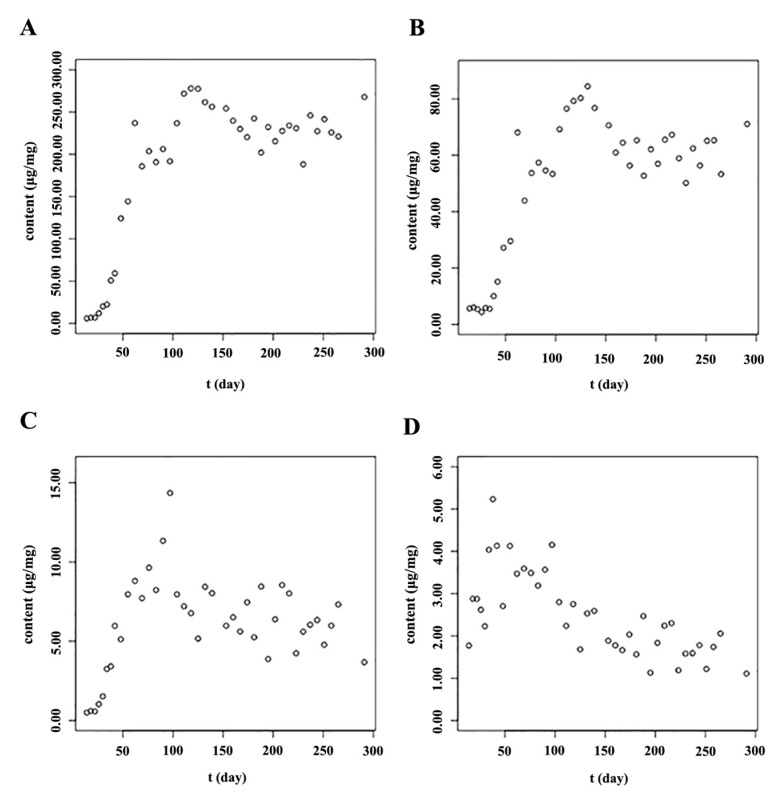
Accumulation patterns of main flavonoids in the root of *S. baicalensis*. (**A**): Baicalin; (**B**): Wogonoside; (**C**): Baicalein; (**D**): Wogonin; The units “μg/mg” meant the contents of analytes in per mg of freeze-dried samples.

After the true leaves of *S. baicalensis* appeared (on the 14th day), the main flavonoids, such as baicalin, wogonoside and their aglycones, accumulated rapidly until to the full-bloom stage. However, with the growth and development of *S. baicalensis*, the contents of isocarthamidin-7-*O-β*-d-glucuronide, scutellarin, carthamidin-7-*O*-*β*-d-glucuronide, apigenin-7-*O*-*β*-d-glucuronide and chrysin-7-*O*-*β*-d-glucuronide in the root were too low to be detected. Therefore, these five flavonoid compounds were not quantified in the root of *S. baicalensis*. As shown in Figure 5, the content of baicalein in the root increased from the 14th day and reached its highest level (14.35 μg/mg) near the full-bloom stage. Then it decreased significantly. The accumulation pattern of wogonin in the root was similar way to that of baicalein. The content of wogonin in the root reached to the highest level (5.23 μg/mg) at the beginning of the reproductive stage (Table S2).

**Figure 6:**
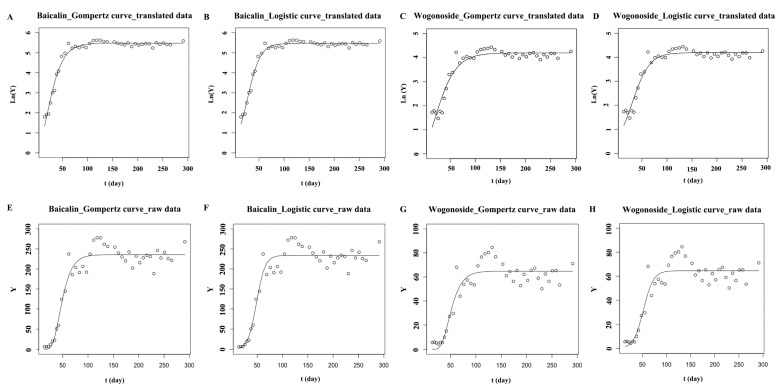
The growth curves fitting for baicalin and wogonoside in root of *S. baicalensis*. (**A**,**C**): the Gompertz growth curves fitted by the translated data of the content of baicalin and wogonoside; (**B**,**D**): the Logistic growth curves fitted by the translated data of the content of baicalin and wogonoside; (**E**,**G**): the Gompertz growths curve fitted by the raw data of the content of baicalin and wogonoside; (**F**,**H**): the Logistic growth curves fitted by the raw data of the content of baicalin and wogonoside; Y meant the contents of the quantified compounds in freeze-dried samples; Ln(Y) meant that the raw data of contents were translated by taking logarithm.

The contents of baicalin and wogonoside in the root accumulated rapidly from when the true leaves appeared. When near to the full-bloom stage, the contents of both baicalin and wogonoside reached their peak values, 277.90 and 84.47 μg/mg, respectively. As baicalin and wogonoside were synthesized from baicalein and wogonin, respectively, when the reproductive organs began to develop, the contents of baicalein and wogonin in the root decreased. As there might not be sufficient aglycones synthesized at the reproductive growth stage, the contents of baicalin and wogonoside stopped increasing and changed on a small scale after the full-bloom stage. The logistic curve and Gompertz curve are commonly used to interpret the progress of biological growth phenomena [37,38]. Both these growth models gave “S”-shaped curves. In the roots of *S. baicalensis*, the baicalin and wogonoside accumulated rapidly in a short time at the initial stage (Figure 5). After that, their contents stopped increasing and fluctuated in a narrow range. The accumulation patterns of baicalin and wogonoside were like “S” curves, so the logistic growth curve and the Gompertz growth curve were used to fit the accumulation patterns of baicalin and wogonoside. Raw data of the contents were transformed into their logarithmic values and also were used to fit the models. As shown in Table 3 and Figure 6, the models matched with the translated data had bigger determination coefficient (R^2^) and smaller sum of squares due to error (SSE) values than those models matched with the raw data. The logistic equation fitted by the translated data showed bigger R^2^ and smaller SSE than the Gompertz equation, indicating that the logistic growth model fitted the accumulation patterns of baicalin and wogonoside in the root of *S. baicalensis* well. Through the models, the contents of baicalin and wogonoside could be predicted, so these models could be used to guide scientific cultivation of *S. baicalensis* and to predict the optimum harvest stage. In general, the main bioactive ingredients enriched rapidly before the full-bloom stage and then changed to a small extent.

### 2.4. Dynamic Accumulation of Flavonoids in Stems and Leaves of S. baicalensis at Different Growth Stages

**Figure 7:**
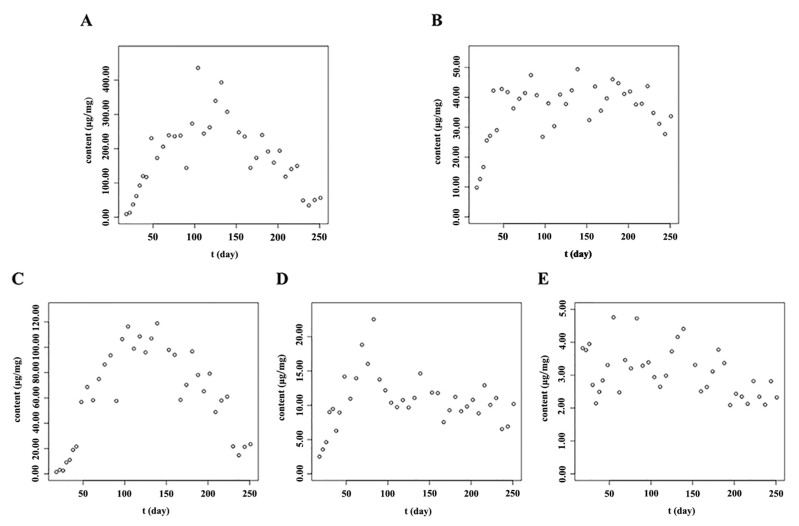
Accumulation patterns of main flavonoids in the leaf of *S. baicalensis*. (**A**): Isocarthamidin-7-*O*-*β*-d-glucuronide; (**B**): Scutellarin; (**C**): Carthamidin-7-*O*-*β*-d-glucuronide; (**D**): Apigenin-7-*O*-*β*-d-glucuronide; (**E**): Baicalin; The units “μg/mg” meant the contents of analytes in per mg of freeze-dried samples.

**Figure 8:**
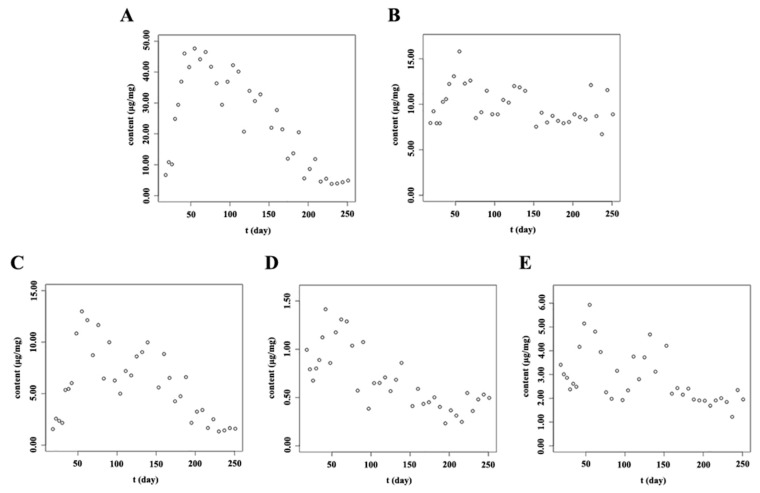
Accumulation patterns of main flavonoids in the stem of *S. baicalensis*. (**A**): Isocarthamidin-7-*O*-*β*-d-glucuronide; (**B**): Scutellarin; (**C**): Carthamidin-7-*O*-*β*-d-glucuronide; (**D**): Apigenin-7-*O*-*β*-d-glucuronide; (**E**): Baicalin; The units “μg/mg” meant the contents of analytes in per mg of freeze-dried samples.

As the results in Figure 7 and Figure 8 and Table S2 indicate, the five main flavonoids—isocarthamidin-7-*O*-*β*-d-glucuronide, scutellarin, carthamidin-7-*O*-*β*-d-glucuronide, apigenin-7-*O*-*β*-d-glucuronide and baicalin—had similar accumulation patterns in the stems and leaves of *S. baicalensis*. The main flavonoids accumulated rapidly after the true leaves appeared. As flavonoids play an important role in UV protection [39,40], they are often presented in the epidermal cell layers of the leaf and in the stem tissues which were susceptible to UV light to protect them from photo-oxidative damage [41]. The reproductive stage (from day 69 to day 167) ranges from June to August. During the reproductive stage, the sunshine duration is the longest and the illumination intensity reached its highest value, so the contents of flavonoids also reach to their highest levels from June to August. However, during the full bloom stage (approximately from day 90 to day 111), the contents of flavonoids in the stems and leaves decreased to some extent. This might be due to the fact that, at the full-bloom stage, flavonoids might be mainly synthesized for reproductive growth and the syntheses of flavonoids in vegetative organs might be depressed, so the contents of flavonoids in stems and leaves decreased to some extent during the full-bloom stage. Therefore, the accumulation patterns of main flavonoids in stems and leaves of *S. baicalensis* showed an “M” shape. Based on the results, the optimum harvest time of stems and leaves are the initial or later phases of the reproductive stage.

### 2.5. Dynamic Accumulation of Flavonoids in Reproductive Organs of S. baicalensis at Different Growth Stages

**Figure 9:**
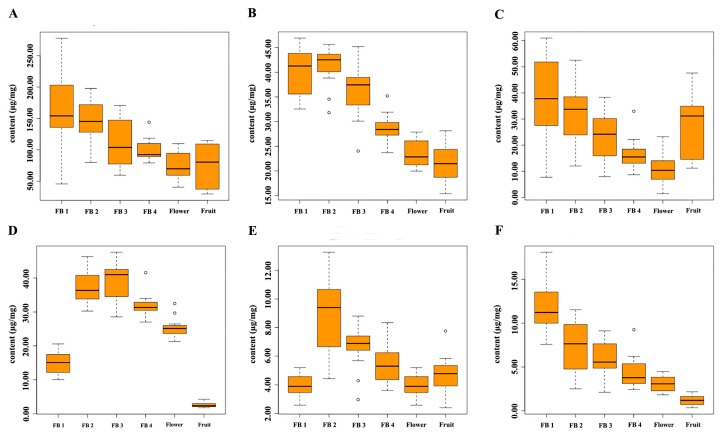
The accumulation patterns of main flavonoids in reproductive organs of *S. baicalensis*. (**A**): Isocarthamidin-7-*O*-*β*-d-glucuronide; (**B**): Scutellarin; (**C**): Carthamidin-*7*-*O*-*β*-d-glucuronide; (**D**): Apigenin-7-*O*-*β*-d-glucuronide; (**E**): Baicalin; (**D**): Chrysin-7-*O*-*β*-d-glucuronide; The units “μg/mg” meant the contents of analytes in per mg of freeze-dried samples. Each “。” meant the content of the analyte in a sample which was bigger than the 75% quantile plus one and a half times of the box length or smaller than the 25% quantile minus one and a half of times of the box length. The box length was the 75% quantile of the content of the analyte in a sample minus the 25% quantile.

The morphological characteristics of the reproductive organs changed with their growth and development. Accompanying the changes in morphological characteristics, the contents of chemical components in the reproductive organs also changed significantly [42,43]. Thus, the accumulation patterns of main flavonoids in the reproductive organs of *S. baicalensis*, isocarthamidin-7-*O*-*β*-d-glucuronide, scutellarin, carthamidin-7-*O*-*β*-d-glucuronide, apigenin-7-*O*-*β*-d-glucuronide, baicalin and chrysin-7-*O*-*β*-d-glucuronide were detected. As the results shown in Figure 9 and Table S2, with the morphological changes, the contents of the main flavonoids in reproductive organs changed significantly during the six different development stages. The contents of main flavonoids reached to their highest levels in FB1, except for apigenin-7-*O*-*β*-d-glucuronide and baicalin. The contents of isocarthamidin-7-*O*-*β*-d-glucuronide and carthamidin-7-*O*-*β*-d-glucuronide reached to their peak values at the initial development stage of the flower buds, and then decreased to their lowest levels during flowering. After that, the contents of these two compounds increased slightly. In flavonoids metabolic pathway, flavanone was the precursor substance of anthocyanin. When flowering, the common precursors might be used to synthesize anthocyanin. This might explain why the contents of isocarthamidin-7-*O*-β-d-glucuronide and carthamidin-7-*O*-β-d-glucuronide decreased with the flower bud growth.

The contents of scutellarin and chrysin-7-*O*-*β*-d-glucuronide decreased continuously during the reproductive organ growth (from FB1 to Fruit). The contents of apigenin-7-*O*-*β*-d-glucuronide increased from FB1 to FB3, then decreased to the lowest level at fruiting. The content of baicalin reached a peak value in FB2 and then decreased to the lowest level when flowering. At the initial flower bud development stage, anthocyanins in the reproductive organs might be not enough to defend against UV damage, so flavones were synthesized to absorb UV [44,45]. As anthocyanins and flavone are synthesized in the same pathway, they share the same precursor substances. Therefore, with the flower bud growth, anthocyanins were synthesized continuously and the synthesis of flavones such as apigenin-7-*O-β*-d-glucuronide, chrysin-7-*O*-*β*-d-glucuronide, scutellarin, baicalin was depressed. In conclusion, the flower budding stages of *S. baicalensis* might be a more optimal time to harvest than the flower and fruit stage when using for medicinal properties and the flower buds could be separated into different grades by length.

## 3. Materials and Methods

### 3.1. Plant Materials and Sample Preparation

The seeds of *S. baicalensis* were collected from Kushan County (Rizhao, China) and planted in the Medicinal Botanics Garden of China Pharmaceutical University (Nanjing, China) in March of 2016. From seed cultivation (day 0) to seedling growth without true leaves (day 12), the whole plants were harvested every 2 days. From the 14th day (day 14) to the 42th day (day 42), the samples were harvested every 4 days. After day 42, the samples were collected every 7 days until the 251th day (day 251). The samples were collected from the beginning of seed cultivation until the aerial parts died. After the first pair of true leaves appeared (day 14), the whole plant was separated into different parts (such as root, stem, leaf, flower bud, flower and fruit). The flower buds were also divided into four stages by length which were flower bud 1(FB1, <0.5 cm), flower bud 2 (FB2, 0.5–1 cm), flower bud 3 (FB3, 1–1.5 cm) and flower bud 4 (FB4, 1.5–2 cm). At each collection time, 10 plants were collected and pooled. All the collected fresh plant materials were flash frozen in liquid nitrogen. The frozen samples were quickly ground to a fine powder and freeze-dried, then stored in −80 °C until analyzed. The samples collected at day 0, day 2, day 4, day 6, day 8, day 10, day 12, day 14, day 18, day 22, day 62 and day 83 were used for qualitative analysis. All the samples collected as mentioned above from day 0 to day 251 were used for quantitative analysis. For quantitative analysis, each freeze-dried mixed sample (from day 0 to day 251) was performed in triplicate, and triplicate samples were extracted, respectively. The accurately weighed freeze-dried powder (5 mg) was suspended in 70% methanol (*v*/*v*, 1 mL), sonicated for 30 min, and then cooled to room temperature. After centrifugation at 12,000 g for 10 min, the supernatant was transferred to a 2 mL volumetric flask. This step was repeated one time and then the volume was adjusted to the calibration mark with 70% methanol (*v*/*v*) [6]. The supernatant solution was filtered through a 0.22 μm filter before analysis. 

### 3.2. Chemicals and Reagents

The reference standards, isocarthamidin-7-*O-β*-glucuronide and carthamidin-7-*O*-*β*-d-glucuronide, were isolated in our laboratory. Baicalin, baicalein, wogonoside, wogonin, apigenin-7-*O*-*β*-d-glucuronide, chrysin-7-*O*-*β*-d-glucuronide, and scutellarin samples were purchased from Chengdu Mansite Biological Technology Co. (Chengdu, China). The purity of each compound was determined to be over 98% by HPLC analysis. Chromatography grade acetonitrile was purchased from Merck (Darmstadt, Germany). Ultrapure water for chromatography was obtained from an ULUP-II-20T purification system (ULUP, Nanjing, China). All other reagents were of analytical grade and purchased from Nanjing Chemical Regents Co. Ltd. (Nanjing, China).

### 3.3. UHPLC-QTOF-MS/MS Based Qualitative Analysis and HPLC Quantification

Chromatographic separation was performed on a Shimadzu LC-30A Series UHPLC system (Shimadzu, Duisburg, Germany). An ultimate HPLC XB-C18 column (150 mm × 2.1 mm, 3 μm, Welch, Shanghai, China) was applied for all analyses at 30 °C. The mobile phase was a mixture of 0.1% formic acid-water (A) and 0.1% formic acid-acetonitrile (B) at a flow rate of 0.3 mL/min. The gradient condition was as follows: 0–6 min, 13% B; 6–8 min, 13% B→18% B; 8–14 min, 18%; 14–15 min, 18% B→23% B; 15–22 min, 23% B; 22–24 min, 23% B→25% B; 24–32 min, 25% B; 32–34 min, 25% B→35% B; 34–39 min, 35% B; 39–42 min, 35% B→40% B; 42–47 min, 40% B; 47–50 min, 40% B→95% B; 50–55 min, 95% B; 55–56 min 95% B–13% B, followed by 1 min of re-equilibration. The injection volume was 4 μL.

Mass spectrometry experiments were accomplished on an AB SCIEX Triple TOF^TM^ 5600+ system (AB SCIEX Technologies, Redwood City, CA, USA) equipped with an electrospray ionization (ESI) source. Samples were analyzed in negative ion modes to provide information for structural identification. The parameters were as follows: mass range, *m*/*z* 100 to 1000; electrospray ionization temperature (°C): 500; nebulizer gas pressure (psi): 60; ion spray voltage (KV): 4.5; collision energies (V): 40.

**Figure 10:**
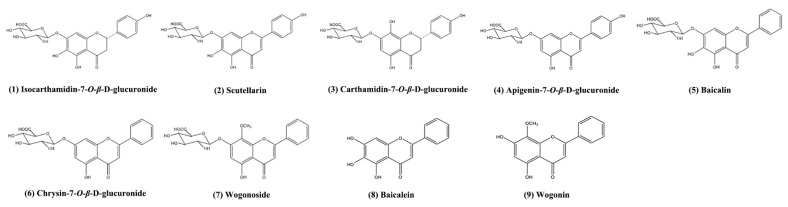
Structures of the quantified compounds. (**1**): Isocarthamidin-7-*O-β*-d-glucuronide; (**2**) Scutellarin; (**3**): Carthamidin-7-*O*-*β*-d-glucuronide; (**4**): apigenin-7-*O*-*β*-d-glucuronide; (**5**): Baicalin; (**6**): chrysin-7-*O*-*β*-d-glucuronide; (**7**): Wogonoside; (**8**): Baicalein; (**9**): Wogonin.

Each sample was quantified for 9 standard substances (Figure 10) using high performance liquid chromatography (HPLC) on an Agilent Series 1260 LC instrument (Agilent Technologies, Cambridge, MA, USA). The chromatographic condition was same as above mentioned and the detection wavelength was at 278 nm [46]. Standard stock solutions of the nine standard substances were prepared and then diluted to appropriate concentrations for calibration and method validation.

### 3.4. Method Validation

Analyses of linear regression curve, limit of detection (LOD), limit of quantification (LOQ), repeatability, intra-day and inter-day stability as well as recovery for each standard substance were performed. The mixed standard solution was diluted with methanol to yield a series of standard solutions at appropriate concentrations to construct the calibration curves. LOD and LOQ were determined with signal-to-noise (S/N) ratios of 3 and 10 respectively.

To evaluate the precision, we analyzed the standard solutions with six replicates, three replicates for intra-day variability and three ones for inter-day variability. The RSD of each standard compounds was calculated. To confirm the repeatability, six different sample solutions were prepared from the same samples (the root which was collected in the 180th day and flower which was collected in the 139th day) were analyzed and variations were expressed by RSD. The stability was evaluated by storing the sample solutions (root which was collected in the 180th day and flower bud 3 which was collected in the 139th day) at 25 °C, then analyzed at 0 h, 2 h, 4 h, 6 h, 8 h, 12 h, 24 h, 48 h and 72 h respectively. As for the recovery validation, six duplicates of root (collected in the 180th day) were added with certain amounts of standard compounds (baicalin, baicalein, wogonoside and wogonin) for extraction and recovery assessment. Six duplicates of flower bud 3 (collected at the 139th day) were added with certain amounts of standard compounds (isocarthamidin-7-*O*-*β*-d-glucuronide, scutellarin, carthamidin-7-*O*-*β*-d-glucuronide, apigenin-7-*O-β*-d-glucuronide, chrysin-7-*O*-*β*-d-glucuronide) for extraction and recovery assessment.

### 3.5. Data Analysis

The raw data from the UPLC-QTOF-MS/MS were qualitatively analyzed by Peakview Software (version1.2.0.3, AB SCIEX, Redwood City, CA, USA). In quantitative analysis, each sample was performed in triplicate, and the data were presented as the mean value ± standard deviation (SD). The units “μg/mg” meant the contents of analytes in per mg of freeze-dried samples.

Gompertz curve and Logistic curve were adopted to describe the relationship between flavonoid contents and growing time. The results were fitted to the models as follows (Equation (1)):

The Gompertz curve: Y = a × exp[−exp (b − c × x)]

The logistic curve: Y = a/[1 + b × exp(−c × x)]
(1)

where Y represented the contents of baicalin or wogonoside. The x was the time after seed cultivation. The a, b, and c were three arbitrary constans, which correspond essentially to the upper asymptote, the time origin, and the time unit [37]. Nonlinear regression analysis was performed in IBM SPSS Statistics 23 (IBM, New York, NY, USA). The other data analysis and their visualization were processed in the software R (version 3.2.4, a language and environment for statistical computing. R Foundation for Statistical Computing, Vienna, Austria.).

## 4. Conclusions

In this study, the metabolic profiles of *S. baicalensis* at different growth stages were characterized by UHPLC-QTOF-MS/MS and HPLC-DAD. The metabolic profiles and quantitative analysis revealed that the chemical compositions in seeds and seedlings were very different from that in mature plants of *S. baicalensis* and there was clear chemical specificity among different organs. At the initial seed germination stage, *S. baicalensis* mainly contained flavonols. With further growth and development, the flavonoids, considered the main bioactive compounds in *S. baicalensis*, were synthesized and accumulated. The study also provided an effective reference for the optimal harvest time according to the dynamic accumulation patterns of target components. It is recommended that the optimal harvest time for the aerial parts of *S. baicalensis* is at the initial stage of reproductive growth and the flower buds should be harvested before flowering for better quality of their medicinal resources. Our study could effectively promote the scientific cultivation and rational utilization of *S. baicalensis*.

## Figures and Tables

**Table 1:** Compounds identified by UHPLC-QTOF-MS/MS in *S. baicalensis* collected at different growth stages.

No.	t_R_(min)	Molecular Formula	[M − H]^−^ Predicted	[M − H]^−^ Measured	Error (ppm)	(−)-ESI-MS/MS Fragment Ions (*m*/*z*)	Identification [Reference]
**1**	1.59	C_21_H_22_O_13_	481.09876	481.09832	−0.9	463.0880, 319.0452, 301.0345, 257.0457, 233.0465, 215.0337, 193.0141, 175.0039,151.0043, 125.0254	Dihydromyricetin-3′-*O*-glucoside [17]
**2**	2.24	C_21_H_22_O_13_	481.09876	481.09866	−0.2	301.0349, 257.0448, 215.0344, 193.0142, 175.0045, 151.0045, 125.0254, 137.0251	Isomer of dihydromyricetin-3′-*O*-glucoside
**3**	2.43	C_21_H_22_O_12_	465.10385	465.10158	−4.9	303.0398, 285.0396, 273.0295, 241.0461, 219.0260, 177.0181, 125.0267	Taxifolin-7-*O*-glucoside [18]
**4**	3.5	C_21_H_22_O_12_	465.10385	465.10313	−1.6	303.0505, 285.0398, 275.0551, 259.0600, 241.0511, 217.0509, 125.0254	Isomer of Taxifolin-7-*O*-glucoside
**5**	4.43	C_21_H_22_O_12_	465.10385	465.10323	−1.3	303.0504, 285.0388, 241.0522, 199.0364, 179.0001, 149.0248, 125.0243	Isomer of Taxifolin-7-*O*-glucoside
**6**	4.84	C_15_H_12_O_9_	335.04086	335.04074	−0.4	183.0294, 169.0135, 139.0404, 125.0252, 115.0405	Methyl digallate [19]
**7**	5.27	C_21_H_22_O_13_	481.09876	481.09846	−0.6	463.0893, 301.0352, 283.0247, 257.0551, 255.0291, 215.03.52, 193.0142, 162.0359, 153.0198, 125.0253	Isomer of dihydromyricetin-3′-*O*-glucoside
**8**	5.69	C_22_H_24_O_12_	479.11950	479.11847	−2.2	317.0648, 285.0444, 165.0204	3,5,7,4′-Tetrahydroxy-6-methoxy Flavanone 3-*O*-*β*-d-glucoside
**9**	7.75	C_15_H_12_O_8_	319.04594	319.04568	−0.8	233.0475, 215.0341, 193.0138, 175.0034, 165.0189, 151.0037, 137.0246, 125.0253	Dihydromyricetin [20]
**10**	8.40	C_21_H_20_O_13_	479.08311	479.08288	−0.5	317.0294, 289.0319, 287.0181, 243.0296, 178.9981, 151.0033, 137.0250	Myricetin-3-*O*-Galactoside [21]
**11**	8.74	C_21_H_20_O_11_	447.09329	447.09334	0.1	285.0460, 217.0147,151.0035	5,7,2′,6′-Tetrahydroxy Flavone 2′-*O*-*β*-d-glucoside [6]
**12**	9.05	C_15_H_12_O_7_	303.05103	303.05052	−1.7	125.0256, 149.0231, 177.0176, 267.0288	3,5,7,2′,6′-Pentahydroxy Flavanone [6]
**13**	9.17	C_21_H_22_O_12_	465.10385	465.10367	−0.4	303.0505, 285.0408, 275.0556, 259.0609, 241.0498, 217.0511, 199.0389, 177.0196, 125.0250	Isomer of Taxifolin-7-*O*-glucoside
**14**	9.4	C_15_H_12_O_8_	319.04594	319.04571	−0.7	215.0349, 193.0139, 175.0037, 165.0189, 151.0039, 137.0249, 125.0249	Isomer of dihydromyricetin
**15**	9.63	C_27_H_30_O_17_	625.14102	625.14242	2.2	463.0871, 300.0274, 271.0245, 255.0309, 243.0309, 178.9969	Quercetin-4′-*O*-*β*-d-glucopyranosyl-(1→2)-*β*-d-glucopyranoside [22]
**16**	9.67	C_21_H_20_O_13_	479.08311	479.08305	−0.1	303.0506, 285.0384, 181.0138, 166.9992, 135.0463, 113.0277	5,6,7,3′,4′-Pentahydroxy Flavanon 7-*O*-*β*-d-glucoronide [12]
**17**	9.88	C_26_H_28_O_14_	563.14063	563.13869	−3.4	503.1135, 473.1102, 443.1020, 383.0779, 353.0672	Schaftoside [15]
**18**	9.92	C_26_H_28_O_14_	563.14063	563.14154	1.6	503.1244, 473.1127, 443.0978, 383.0791, 353.0662	Isoschaftoside [15]
**19**	9.98	C_21_H_20_O_11_	447.09329	447.09289	−0.9	285.0403, 284.0319, 240.0448, 227.0302, 228.0412	Kaempferol-3-*O*-glucoside [23]
**20**	10.29	C_21_H_22_O_11_	449.10894	449.10785	−2.4	125.0252, 151.0029, 161.0244, 177.0204, 227.0360, 269.0466, 287.0558	5,7,2′,6′-TetrahydroxyFlavanone 2′-*O*-*β*-d-glucoside [6]
**21**	10.46	C_22_H_22_O_12_	477.10385	477.10258	−2.7	301.0349, 283.0264, 164.9816	6-Hydroxyluteolin 7-*O*-*β*-d-glucoronide [6]
**22**	11.04	C_21_H_22_O_12_	465.10385	465.10384	0	303.0872, 285.0765, 259.0977, 244.0734, 137.0611	Isomer of Taxifolin-7-*O*-glucoside
**23**	11.16	C_27_H_30_O_16_	609.14611	609.14694	1.4	489.1024, 447.0911, 429.0817, 284.0321, 255.0295, 227.0340, 211.0394, 178.9980, 151.0031	Unknown
**24**	12.13	C_26_H_28_O_13_	547.14571	547.14391	−3.3	487.1257, 457.1136, 427.1046, 367.0819, 337.0717	Chrysin 6-*C*-arabinoside-8-*C*-glucoside [7]
**25**	12.66	C_21_H_20_O_12_	463.08820	463.08660	−3.5	287.0560, 269.0541, 259.0602, 181.0142, 166.9993, 153.0201, 119.0517	Isocarthamidin -7-*O-β*-d-glucuronide [24]
**26**	12.91	C_21_H_18_O_12_	461.07255	461.07073	−3.9	285.0395, 257.0435, 164.9827, 136.9883, 119.0517	Luteolin 7-*O*-glucuronide [25]
**27**	13.27	C_21_H_20_O_11_	447.09329	447.09284	−1	285.0390, 284.0314, 271.0611, 256.0293	Isomer of Kaempferol-3-*O*-glucoside
**28**	13.47	C_23_H_24_O_13_	507.11441	507.11317	−2.4	345.0617, 330.0379, 315.0150	Viscidulin III 6′-*O*-*β*-d-glucoside [6]
**29**	13.59	C_21_H_18_O_12_	461.07255	461.07162	−2.0	285.0401, 267.0292, 239.0363, 213.0548, 195.0455	Scutellarin [24]
**30**	14.00	C_29_H_36_O_15_	623.19814	623.19657	−2.5	461.1696, 161.0239	Acteoside [6]
**31**	14.13	C_26_H_28_O_13_	547.14571	547.14518	−1.0	457.1140, 427.1040, 367.0822, 337.0720	Chrysin 6-*C*-glucoside-8-*C*-arabinoside [15]
**32**	14.84	C_21_H_20_O_12_	463.08820	463.08735	−1.8	287.0542, 269.0453, 259.0616, 193.0147, 181.0147, 166.0021, 153.0203, 119.0520	carthamidin -7-*O*-*β*-d-glucuronide [24]
**33**	15.55	C_23_H_24_O_12_	491.11950	491.11730	−4.5	329.0659, 314.0432, 299.0189	5,2′,6′-Dihydroxy-7,8-dimethoxy Flavone 2′-*O*-*β*-d-glucoside [6]
**34**	17.07	C_21_H_18_O_12_	461.07255	461.07033	−4.8	285.0408, 257.0463, 229.0451, 241.0514, 213.0581, 199.0387, 185.0590, 113.0268	Kaempferol-3-*O*-*β*-d-glucuronide [26]
**35**	17.17	C_21_H_20_O_9_	415.10346	415.10199	−3.5	325.0714, 295.0610, 267.0661	Chrysin 8-*C*-*β*-d-glucoside [6]
**36**	17.25	C_21_H_20_O_10_	431.09837	431.09691	−3.4	269.0447	Apigenin-7-*O-β*-d-glucoside [25]
**37**	17.43	C_22_H_20_O_12_	475.08820	475.08685	−2.8	299.0549, 271.0641, 256.0365, 227.0761, 165.0218	5,6,7-Trihydroxy-8-methoxy-7-*O-β*-d-glucuronide [27]
**38**	17.53	C_21_H_20_O_11_	447.09329	447.09216	−2.5	285.0393, 284.0321, 269.0411, 257.0456, 255.0291, 227.0324, 213.0568, 151.0042, 107.0157	Isomer of Kaempferol-3-*O*-glucoside
**39**	17.64	C_24_H_26_O_13_	521.13006	521.12836	−3.3	359.0769, 344.0544, 329.0311, 314.0057	5,2′,6′-Trihydroxy-6,7,8,-trimethoxy Flavone 2′-*O-β*-d-glucoside [2]
**40**	17.79	C_15_H_10_O_6_	285.04046	285.04014	−1.1	199.0388, 151.0054	5,7,2′,6′-Tetrahydroxy Flavone [6]
**41**	17.90	C_15_H_12_O_6_	287.05611	287.05510	−3.5	259.0661, 177.0546, 125.0245	Carthamidin [25]
**42**	17.98	C_21_H_18_O_11_	445.07764	445.07600	−3.7	269.0462, 225.0563, 113.0261	Apigenin-7-*O-β*-d-glucuronide [12]
**43**	18.07	C_21_H_20_O_11_	447.09239	447.09218	−2.5	271.0604, 177.0223, 151.0043, 119.0516, 113.0261, 107.0160	Naringenin-7-*O-β*-d-glucuronide [7]
**44**	18.29	C_21_H_20_O_12_	463.08820	463.08743	−1.7	287.0570, 269.0420, 259.0658, 193.0149, 181.0149, 166.9982, 153.0196, 139.0041, 119.0531	Eriodictyol-7-*O-β*-d-glucuronide [25]
**45**	18.38	C_15_H_12_O_6_	287.05611	287.05516	−3.3	181.0142, 153.0188, 166.9973, 139.0038, 136.9891, 119.0497	Isocarthamidin [28]
**46**	18.59	C_15_H_10_O_8_	317.03029	317.02999	−1	289.0304, 271.0231, 261.0358, 243.0274, 227.0334, 193.0143, 178.9985, 165.0192, 151.0044, 137.0254, 125.0246, 109.0310, 107.0148	Myricetin [29]
**47**	18.64	C_22_H_20_O_12_	475.08820	475.08692	−2.7	299.0560, 284.0314	5,7,8-Trihydroxy-6-methoxy Flavone-7-*O-β*-d-glucuronide [30]
**48**	18.68	C_21_H_18_O_12_	461.07255	461.07194	−1.3	285.0353, 139.0017, 257.0466, 164.9834, 136.9886, 119.0510	Isoscutellarein 8-*O-β*-d-glucuronide [12]
**49**	18.98	C_22_H_22_O_12_	477.10385	477.10144	−5.0	331.0311, 301.0334, 180.9795, 155.0067, 119.0530	5,7,2′-Trihydroxy-6-methoxy Flavanone 7-*O-β*-d-glucuronoide [15]
**50**	19.01	C_22_H_22_O_12_	477.10385	477.10234	−3.2	301.0697, 286.0475, 181.0148	5,7,2′-Trihydroxy-8-methoxyFlavanone 7-*O-β*-d-glucuronoide [15]
**51**	20.28	C_21_H_20_O_11_	447.09329	447.09211	−2.6	285.0397, 267.0289, 241.0539, 199.0371, 151.0062	Isomer of Kaempferol-3-*O*-glucoside
**52**	20.58	C_16_H_14_O_7_	315.05103	315.05069	−1.1	300.0276, 272.0319, 244.0378, 216.0419, 187.0398, 180.9781, 152.9843, 124.9893	Pedalitin
**53**	20.67	C_15_H_10_O_6_	285.04046	285.04042	−0.1	239.0327, 167.0010, 137.0260, 117.0353	Scutellarein [25]
**54**	21.17	C_23_H_24_O_12_	491.11950	491.11970	0.4	329.0635, 314.0426, 299.0252	5,2′,6′-Dihydroxy-6,7-dimethoxy Flavone 2′-*O-β*-d-glucoside [30]
**55**	21.28	C_21_H_20_O_10_	431.09837	431.09702	−3.1	269.0445, 251.0354, 195.0465, 167.0506	Baicalein 7-*O-β*-d-glucoside [6]
**56**	21.36	C21H18O11	445.07764	445.07622	−3.2	269.0456, 251.0345, 223.0397, 197.0621, 113.0262	Baicalin [12]
**57**	21.70	C_15_H_10_O_5_	269.04555	269.04489	−2.4	251.0376, 241.0485, 223.0416, 195.0458, 139.0061, 111.0072	Islandicin
**58**	22.08	C_15_H_10_O_6_	285.04046	285.03977	−2.4	241.0509, 213.0540, 195.0482, 239.0333	Isoscutellarein [26]
**59**	22.68	C_21_H_18_O_12_	461.07255	461.07031	−4.9	285.0415, 267.0285, 239.0317	Isoscutellarein-7-*O-β*-d-glucuronide [12]
**60**	23.50	C_31_H_40_O_15_	651.22944	651.22685	−4.0	475.1832, 193.0499, 175.0398, 160.0171	Cistanoside D [6]
**61**	24.31	C_22_H_20_O_12_	475.08820	475.08707	−2.4	299.0565, 284.0332	5,7,2′-Trihydroxy-6-methoxy Flavone 7-*O-β*-d-glucuronoide [2]
**62**	24.37	C_21_H_20_O_11_	447.09329	447.09150	−4.0	271.0610, 243.0652, 113.0237	Dihydrobaicalin [6]
**63**	24.41	C_22_H_20_O_12_	475.08820	475.08747	−1.5	460.1019, 299.0550, 297.0401, 284.0195, 282.0195, 254.0195	Isomer of cirsimaritin 4′-glucoside
**64**	24.72	C_16_H_14_O_6_	301.07176	301.07067	−3.6	286.0474, 181.0144, 165.9916, 137.9972, 119.0515, 110.0026	5,7,4′-Trihydroxy-6-methoxy Flavanone [15]
**65**	25.56	C_21_H_18_O_11_	445.07764	445.07576	−4.2	269.0449, 241.0494, 225.0556, 197.0611, 171.0451	Norwogonin 7-*O-β*-d-glucuronide [6]
**66**	25.86	C_22_H_22_O_12_	477.10385	477.10214	−3.6	301.0712, 283.0620, 273.0774, 268.0393, 139.0037, 113.0262	5,7,2′,6′-Tetrahydroxy flavonol 7-*O-β*-d-glucuronoide [26]
**67**	26.22	C_22_H_22_O_11_	461.10894	461.10732	−3.5	299.0536, 284.0328, 171.0449	5,7,2′-Trihydroxy-6-methoxy Flavone 7-*O-β*-d-glucoside [6]
**68**	27.11	C_21_H_18_O_11_	445.07764	445.07556	−4.7	269.0450, 241.0485, 225.0560, 171.0472	Norwogonin 8-*O-β*-d-glucuronide [6]
**69**	27.76	C_21_H_18_O_10_	429.08272	429.08064	−4.8	253.0499, 209.0601,143.0503,113.0260	Chrysin 7-*O-β*-d-glucuronide [24]
**70**	27.96	C_22_H_20_O_11_	459.09329	459.09148	−3.9	283.0612, 268.0376	Oroxylin A 7-*O-β*-d-glucuronide [6]
**71**	28.68	C_22_H_20_O_12_	475.0882	475.08663	−3.3	299.0549, 284.0320	Isomer of cirsimaritin 4′-glucoside
**72**	28.87	C_17_H_14_O_7_	329.06668	329.06548	−3.6	314.0451, 299.0185, 271.0237, 164.9808, 136.9872	5,2′,6′-Trihydroxy-7,8-dimethoxy Flavone [2]
**73**	29.64	C_16_H_14_O_6_	301.07176	301.07086	−3.0	286.0459, 181.0153, 165.9924, 137.9976, 119.0521, 110.0027	5,7,4′-Trihydroxy-8-methoxy Flavanone [15]
**74**	29.84	C_21_H_18_O_11_	445.07764	445.07594	−3.8	269.0455, 251.0341, 223.0383, 113.0273	Baicalein 6-*O-β*-d-glucuronide [6]
**75**	30.6	C_15_H_10_O_6_	285.04046	285.03945	−3.6	151.0030, 107.0150	Luteolin [12]
**76**	30.81	C_22_H_20_O_11_	459.09329	459.09140	−4.1	283.0613, 268.0375	Wogonoside [12]
**77**	31.34	C_23_H_24_O_11_	475.12459	475.12307	−3.2	460.0994, 445.0748, 313.0665, 297.0390, 283.0199, 282.0144, 254.0212, 226.0276, 183.0437	Cirsimaritin 4′-*O*-glucoside [31]
**78**	32.05	C_21_H_20_O_10_	431.09837	431.09633	−4.7	255.0674, 213.0562, 187.0791, 151.0039, 113.0259	5,7-dihydroxy Flavanone 7-*O-β*-d-glucuronoide [12]
**79**	32.65	C_23_H_22_O_12_	489.10385	489.10194	−3.9	313.0712, 298.0485, 283.0243, 255.0361, 211.0416, 113.0274	5,7-Dihydroxy-2′,8-dimethoxy Flavone 7-*O-β*-d-glucuronide [24]
**80**	33.30	C_18_H_16_O_8_	359.07724	359.07546	−4.9	344.0565, 329.0297, 314.0125, 286.0098, 194.9928	5,2′,5′-Trihydroxy-6,7,8-trimethoxy Flavone [2]
**81**	34.84	C_16_H_12_O_6_	299.05611	299.05566	−1.5	284.0323, 255.0232, 227.0358, 211.0477, 183.0436, 164.0130, 136.9906	Isomer of 5,7,4′-Trihydroxy-8-methoxy Flavone
**82**	34.91	C_15_H_10_O_5_	269.04555	269.04478	−2.9	241.0454, 225.0531, 195.0443, 171.0439, 117.0358	Apigenin [24]
**83**	36.07	C_17_H_14_O_7_	329.06668	329.06584	−2.5	314.0461, 299.0201, 227.0325, 165.9927, 137.9951, 110.0020	5,8,2′-Trihydroxy-6,7-dimethoxy Flavone [6]
**84**	36.28	C_17_H_14_O_6_	313.07176	313.07085	−2.9	298.0542, 283.0251, 244.8595, 211.0396, 166.8658, 155.0508	5,8-Dihydroxy-6,7-dimethoxy Flavone [30]
**85**	36.59	C_16_H_12_O_6_	299.05611	299.05552	−2.0	284.0318, 255.0274, 239.0370, 171.0454, 153.9920, 125.9966	5,7,4′-Trihydroxy-8-methoxy Flavone [30]
**86**	37.11	C_18_H_16_O_7_	343.08233	343.08170	−1.8	328.0591, 313.0331, 298.0112, 270.0167, 241.0502, 226.0271, 198.0335, 185.0629, 155.0521	SkullcapFlavone I [27]
**87**	37.16	C_15_H_10_O_5_	269.04555	269.04510	−1.7	251.0348, 241.0499, 223.0399, 195.0442, 169.0660, 136.9880	Baicalein [6]
**88**	37.54	C_15_H_12_O_5_	271.06120	271.06073	−1.7	243.0638, 225.0535, 185.0601, 152.0182, 139.0070, 124.0182	Dihydronorwogonin [2]
**89**	37.67	C_17_H_14_O_7_	329.06668	329.06602	−2.0	314.0367, 299.0139	5,7,2′-Trihydroxy-8,6′-dimethoxy Flavone [32]
**90**	38.30	C_16_H_12_O_6_	299.05611	299.05531	−2.7	284.0312, 165.9925	5,6,7-Trihydroxy-4′-methoxy Flavone [33]
**91**	44.26	C_18_H_16_O_7_	343.08233	343.08158	−2.2	328.0584, 313.0347, 298.0130, 285.0400, 164.9831	SkullcapFlavone [6]
**92**	44.65	C_16_H_12_O_5_	283.06120	283.06097	−0.8	268.0371, 239.0353, 211.0401, 184.0532, 163.0037, 110.0021	Wogonin [6]
**93**	45.47	C_15_H_10_O_4_	253.05063	253.05033	−1.2	209.0616, 166.8665, 143.0516, 107.0165	Chrysin [24]
**94**	46.31	C_19_H_18_O_8_	373.09289	373.09147	−3.8	358.0616, 343.0434, 328.0199, 300.0248	5,6′-Dihydroxy-6,7,8,2′-tetramethoxy Flavone [6]
**95**	46.59	C_16_H_12_O_5_	283.06120	283.06056	−2.3	268.0373, 239.0358, 211.0363, 184.0525, 110.0016	Oroxylin A [6]
**96**	46.66	C_15_H_12_O_4_	255.06628	255.06670	1.6	213.0545, 201.8339, 182.9022, 166.8653	Pinocembrin [12]
**97**	47.20	C_17_H_14_O_6_	313.07176	313.07047	−4.1	298.0505, 283.0256, 255.0307, 183.0454, 164.9835	5,7-Dihydroxy-6,8-dimethoxy Flavone [27]
**98**	49.45	C_18_H_16_O_7_	343.08233	343.08130	−3.0	328.0598, 313.0346, 298.0124, 270.0161	Tenaxin I [6]

**Table 2:** The unique compounds identified at different growth stages and in different organs.

	Number of the Compound
**Different growth stage**	
Seed stage	**83**
Seedling stage	**37**, **59**, **67**, **72**, **75**, **77**, **80**, **97**
Mature stage	**3**, **8**, **48**, **88**
**Different Organ**	
Root	**11**, **12**, **18**, **28**, **35**, **39**, **70**, **74**, **79**, **85**, **91**, **98**
Stem	**60**, **78**
Leaf	**40**
Reproductive organs	**2**, **3**, **4**, **6**, **7**, **8**, **10**, **11**, **12**, **14**, **15**, **23**, **36**, **38**, **41**, **46**, **50**, **51**, **96**

The numbering of compounds is the same as in Table 1.

**Table 3:** Growth models for the accumulation of baicalin and wogonoside in root of *S.baicalensis*.

Model	Equation	Chem	Parameter Values				
			a	b	c	R^2^	SSE
Gompertz	Y = a × exp[−exp(b − c × t)]	Baicalin	235.492	3.303	0.076	0.936	19,527.371
		Baicalin *	5.485	1.172	0.058	0.977	1.172
		Wogonoside	67.777	3.246	0.07	0.877	2928.026
		Wogonoside *	4.205	0.896	0.043	0.923	2.609
Logistic	Y = a/[1 + b × exp(−c × t)]	Baicalin	233.686	366.795	0.12	0.933	20,164.7
		Baicalin *	5.468	8.437	0.078	0.984	0.816
		Wogonoside	64.517	171.301	0.098	0.878	2902.031
		Wogonoside *	4.189	5.864	0.058	0.938	2.102

Y meant the contents of the quantified compound and t means the growth time of *S. baicalensis*. * The raw data of contents were translated by taking logarithm.

## Supplementary Materials

The following are available online. Figure S1: The structures of the identified compounds; Table S1: The method validation results; Table S2: The contents of quantified compounds in the roots, stems, leaves of *S. baicalensis*; Table S3: The contents of quantified compounds in the reproductive organs of *S. baicalensis*.
