# Supplementary material for: Organ-Specific Metabolic Shifts of Flavonoids in Scutellaria baicalensis at Different Growth and Development Stages
Source: Molecules. 2018 Feb 15;23(2):428. doi: 10.3390/molecules23020428 (PMC6017826; doi:10.3390/molecules23020428)
Supplement: Supplementary file 1 [file molecules-23-00428-s001.zip › Figure S1.pdf]

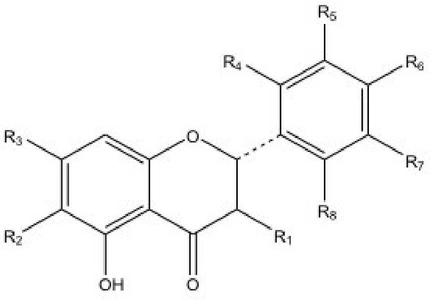

Flavanonol

No.	R1	R2	R3	R4	R5	R6	R7	R8
1	OH	H	OH	H	Oglu	OH	OH	H
3	OH	H	Oglu	H	OH	OH	H	H
8	Oglu	OCH3	OH	H	H	OH	H	H
9	OH	H	OH	H	OH	OH	OH	H
12	OH	H	OH	OH	H	H	H	OH

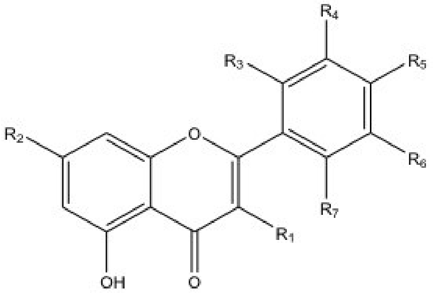

Flavonol

No.	R1	R2	R3	R4	R5	R6	R7
10	Ogal	OH	H	OH	OH	OH	H
15	OH	OH	H	H	Oglu-Oglu	OH	H
19	Oglu	OH	H	H	OH	H	H
34	OgluA	OH	H	H	OH	H	H
46	OH	OH	H	OH	OH	OH	H
66	OH	OgluA	OH	H	H	H	OH

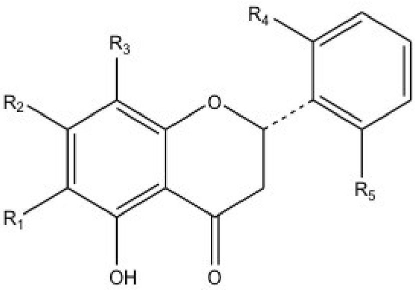

Flavanone without 4’-OH

No.	R1	R2	R3	R4	R5
20	H	OH	H	Oglu	OH
49	OCH3	OgluA	H	OH	H
50	H	OgluA	OCH3	OH	H
62	OH	OH	H	H	H
78	H	OgluA	H	H	H
79	H	OgluA	OCH3	OCH3	H
88	H	OH	OH	H	H
96	H	OH	H	H	H

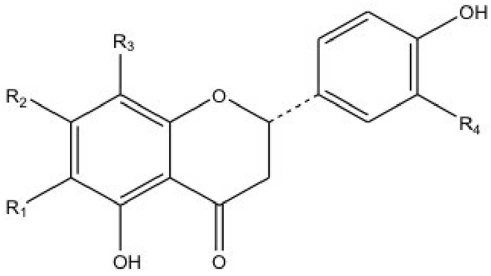

Flavanone with 4’-OH

NO.	R1	R2	R3	R4
16	OH	OgluA	H	OH
25	OH	OgluA	H	H
32	H	OgluA	OH	H
41	H	OH	OH	H
43	H	OgluA	H	H
44	H	OgluA	H	OH
45	OH	OH	H	H
73	H	OH	OCH3	H

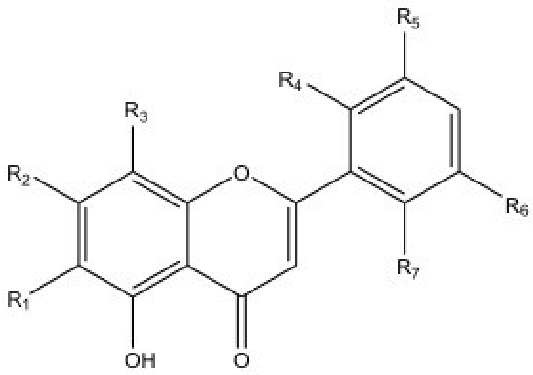

Flavone without 4'-OH

No.	R1	R2	R3	R4	R5	R6	R7
11	H	OH	H	Oglu	H	H	OH
24	Ara	H	Glu	H	H	H	H
28	H	OH	OCH3	OCH3	OH	H	Oglu
31	Ara	H	Glu	H	H	H	H
33	H	CH3	OCH3	Oglu	H	H	OH
35	H	H	Glu	H	H	H	H
37	OH	Oglu	OCH3	H	H	H	H
39	OCH3	CH3	OCH3	Oglu	H	H	OH
40	H	H	H	OH	H	H	OH
47	OCH3	OgluA	OH	H	H	H	H
54	OCH3	OCH3	H	OgluA	H	H	OH
55	OH	Oglu	H	H	H	H	H
56	OH	OgluA	H	H	H	H	H
61	OCH3	OgluA	H	OH	H	H	H
65	H	OgluA	OH	H	H	H	H
67	OCH3	OgluA	H	OH	H	H	H
68	H	OH	OgluA	H	H	H	H

No.	R1	R2	R3	R4	R5	R6	R7
11	H	OH	H	Oglu	H	H	OH
24	Ara	H	Glu	H	H	H	H
28	H	OH	OCH3	OCH3	OH	H	Oglu
31	Ara	H	Glu	H	H	H	H
33	H	CH3	OCH3	Oglu	H	H	OH
35	H	H	Glu	H	H	H	H
37	OH	Oglu	OCH3	H	H	H	H
39	OCH3	CH3	OCH3	Oglu	H	H	OH
40	H	H	H	OH	H	H	OH
47	OCH3	OgluA	OH	H	H	H	H
54	OCH3	OCH3	H	OgluA	H	H	OH
55	OH	Oglu	H	H	H	H	H
56	OH	OgluA	H	H	H	H	H
61	OCH3	OgluA	H	OH	H	H	H
65	H	OgluA	OH	H	H	H	H
67	OCH3	OgluA	H	OH	H	H	H
68	H	OH	OgluA	H	H	H	H

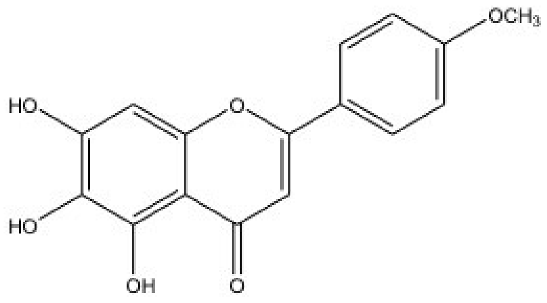

NO. 90 5,6,7-Trihydroxy-4'-methoxy flavone

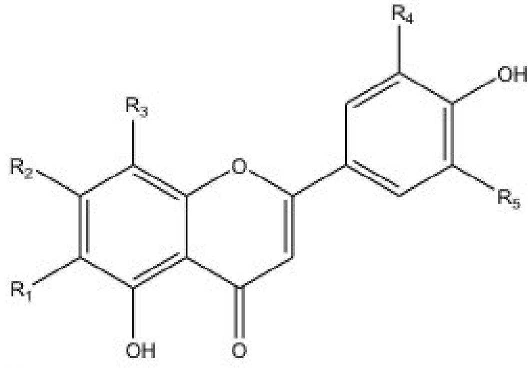

Flavone with 4'-OH

No.	R1	R2	R3	R4	R5
17	Oglu	OH	Oara	H	H
18	Oara	OH	Oglu	H	H
21	OH	OgluA	H	H	OH
26	H	OgluA	H	H	OH
29	OH	OgluA	H	H	H
36	H	Oglu	H	H	H
42	H	OgluA	H	H	H
48	H	OH	OgluA	H	H
52	OH	OCH3	H	OH	H
53	OH	OH	H	H	H
58	H	OH	OH	H	H
59	H	OgluA	OH	H	H
64	OCH3	OH	H	H	H
75	H	OH	H	OH	H
82	H	OH	H	H	H
85	H	OH	OCH3	H	H

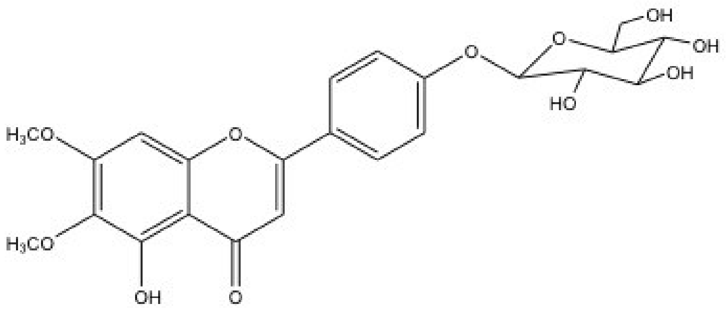

NO. 77 Cirsimaritin 4'-O-glucoside

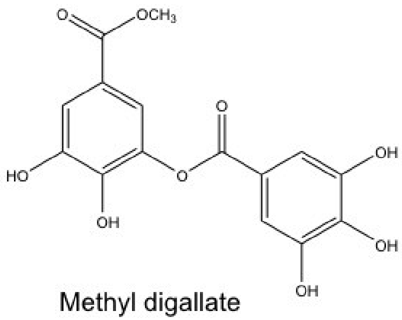

Methyl digallate

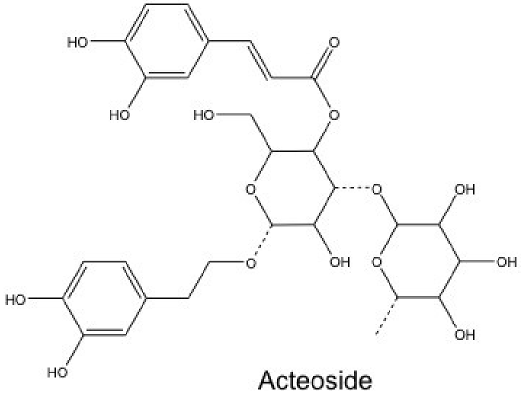

Acteoside

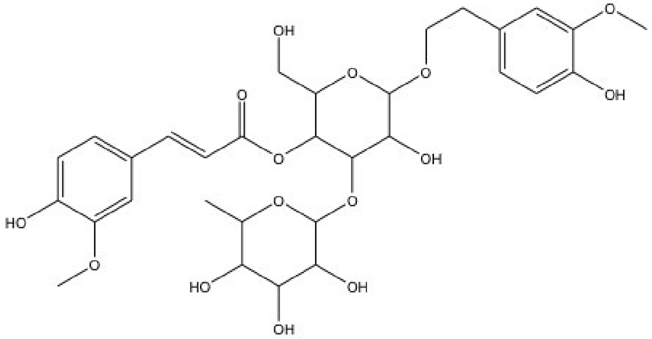

Cistanoside D

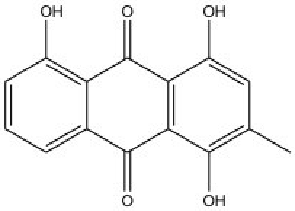

Islandicin

Figure S1 The structures of the identified compounds in *S. baicalensis*

The No. of the compound is the same as it in Table 1.

glu = glucose, Ara = arabinose, glu A= glucuronic acid
